# US & MR/CT Image Fusion with Markerless Skin Registration: A Proof of Concept

**DOI:** 10.1007/s10278-024-01176-w

**Published:** 2024-07-17

**Authors:** Martina Paccini, Giacomo Paschina, Stefano De Beni, Andrei Stefanov, Velizar Kolev, Giuseppe Patanè

**Affiliations:** 1 CNR-IMATI ‘E. Magenes’, via de Marini, 6, Genova, Italy; 2 Esaote S.p.a., via E. Melen, 77, Genova, Italy; 3 MedCom GmbH, Dolivostr., 11, Darmstadt, 64293 Germany

**Keywords:** Computed tomography, Depth camera, Magnetic resonance imaging, Medical image fusion, Medical diagnostic imaging, Image processing, Skin segmentation, Ultrasound

## Abstract

This paper presents an innovative automatic fusion imaging system that combines 3D CT/MR images with real-time ultrasound acquisition. The system eliminates the need for external physical markers and complex training, making image fusion feasible for physicians with different experience levels. The integrated system involves a portable 3D camera for patient-specific surface acquisition, an electromagnetic tracking system, and US components. The fusion algorithm comprises two main parts: skin segmentation and rigid co-registration, both integrated into the US machine. The co-registration aligns the surface extracted from CT/MR images with the 3D surface acquired by the camera, facilitating rapid and effective fusion. Experimental tests in different settings, validate the system’s accuracy, computational efficiency, noise robustness, and operator independence.

## Introduction

Medical imaging offers many image acquisition techniques, which allow us to obtain information related to different tissues with various settings, such as signal-to-noise ratio, contrast, and resolution. Generally, high-resolution imaging requires extended image acquisition time, thus making these techniques unsuitable for real-time image processing and analysis. For instance, surgical tools guidance requires monitoring and guiding the insertion of a biopsy needle. Similarly, in cardiological imaging, ongoing tracking of organ functional reactions is crucial for evaluating heart function. In contrast to high-resolution imaging (e.g., CT, PET, MRI), US imaging allows real-time acquisition. Even if 3D US ultrasound is established in some medical branches [1, 2], in many applications, sonographers still rely on 2D images. US imaging assists physicians in various interventional applications, from simple biopsies to more thorough procedures like mini-invasive tumour treatment or neurosurgery. However, the US has a reduced field of view compared to other imaging techniques and a lower quality of features, such as image resolution or the revelation and reproduction of certain kinds of tissue, e.g., soft tissues. Therefore, a key medical imaging functionality is the combination or fusion of real-time US with other acquisition modalities [3].

**Fig. 1:**
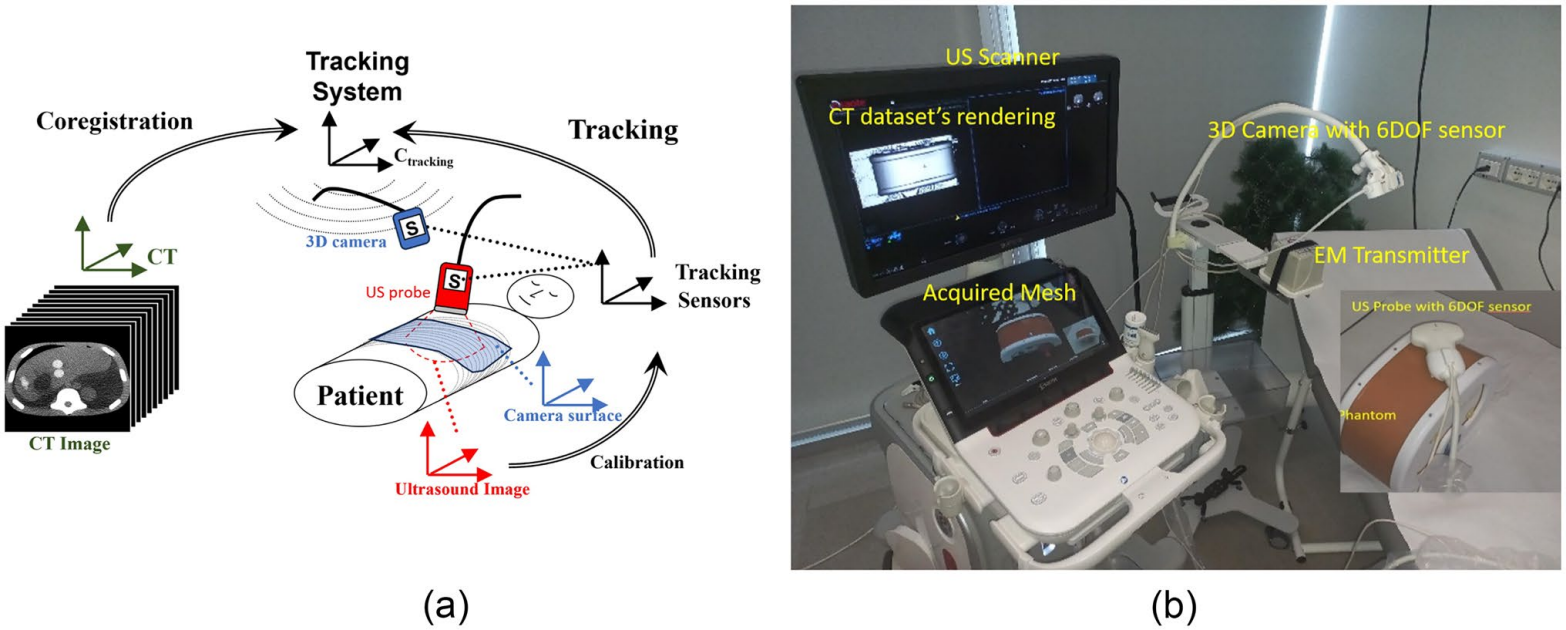
**a** Hardware and software components of the system and their mutual interaction for the image fusion. **b** US system integrated into a testing setup that mimics the clinical environment

This paper introduces an innovative *image fusion system* (Fig. 1) that combines 3D CT/MR images with US acquisition in real-time. Hardware and software components are designed to seamlessly integrate the resulting system into a clinical environment, particularly in interventional radiology, with a direct approach that does not require specific training. This efficient and intuitive integration is achieved through a 3D depth camera able to acquire a 3D surface of the subject undergoing the US exam as quickly as photograph-taking [4]. Indeed, the surface obtained by the 3D camera bridges the 3D anatomy acquired by MR/CT and US images, allowing their fast fusion. The highly portable 3D camera can be introduced in an operating room without compromising the pre-existing set-up. The other hardware components of the image fusion system include a US system and a simplified electromagnetic (EM) tracking system that does not require the placement of physical markers or fiducial patches.

The tracking system comprises an electronic unit, a mid-range transmitter, and sensors for tracking traces of the position and coordinates of the 3D camera and the US probe. The transmitter generates an electromagnetic field with a maximum tracking distance of up to 1800 mm that can simultaneously track the sensors at 70 times per second. The two required sensors are placed on the US probe and the 3D camera to establish their spatial relationship within the fusion imaging setup (Fig. 1). One sensor is connected to the US probe, and the other is associated with the 3D camera, allowing the representation of the US image and the 3D surface acquired by the camera in a unique coordinate system (i.e., the tracking coordinates). The software components can be divided into tracking, surface co-registration, and visualisation software. The system segments the skin surface from the CT/MRI and generates a 3D surface overlay on the patient’s 3D rendering. Then, the clinician acquires the 3D skin with the 3D camera, and the *co-registration software* enables the image fusion within a few seconds, together with the registration error visualisation, facilitating the identification of potential mismatches in the scan area. Upon successful registration, the system presents MR or CT images alongside the US image in various visualisation modes. This allows the clinician to access the corresponding anatomical information and real-time US data during the examination. In particular, the MRI or CT image will move coherently with the US probe during the examination, including changes in the scanning directions.

The proposed system can be applied to different clinical scenarios, such as emergency department, interventional radiology, or any situation where the aligned or superimposed visualisation of the US image and the CT/MRI can provide more information to the physician. The CT/MRI can be either acquired in the same context of the US examination, which could likely be the case of application in emergency departments, or even less recent acquisition, which is expected in the case of interventional radiology (e.g., thermal ablation therapy of the liver tumour) or further investigation exams. Differently from state-of-the-art methods (see the “Related Work’’ section), where the registration is based mainly on the doctor’s ability and requires a long learning curve, the proposed image fusion is suitable even for radiologists with lower experience levels. Moreover, the proposed method avoids external physical markers, which, despite the exciting results [5], could not comply with the existing hospital workflow since marker positioning requires time and can be error-prone. The skin segmentation method developed is highly general and can be applied to different anatomical regions and image types. Even the new AI-based methods for automatic liver shape or vessel tree segmentation require sequences that are only sometimes present in the patient data set and encounter even more complications due to the high variability of the images from series to series. The proposed image fusion system (see the “MR & US Fusion System’’ section) has been tested considering different aspects: the co-registration accuracy between MR/CT and US images (millimetric error), the computational cost for real-time applications (in seconds), noise robustness, and independence from the operator (see the “ Experimental Results and Validation’’ section and “Conclusions and Future Work’’ section).

## Related Work

### Fusion Imaging

Fusion imaging, along with other emerging techniques [6], is in the guideline for several clinical procedures like targeted prostate biopsy that allow more comfort for the patient and more reliable results in the tissue sampling [7, 8]. In abdominal applications, fusion imaging is widely used for liver tumour treatment using ablation techniques based on radiofrequency (RF) needles, microwave (MW) antennas, laser fibre, or cryoprobes. All these techniques require the placement of ablation electrodes or applicators into the lesion and the deployment of energy until the tissue reaches a temperature over 65° (RF MW Laser) or less than −18° (Cryo), causing cellular death. The core concept in fusion imaging is accurately registering the patient’s anatomy in different medical imaging data, such as US, CT, or MR images, which implies aligning different acquisitions into a standard reference system. This process often leads to overcomplicated systems for an actual clinical application. Image fusion is carried out by tracking the US probe’s position, orientation, and displacements during the acquisition within a reference system standard to the US images and the other modalities considered. This setting implies using probe trackers of different natures: probe trackers based on (i) optical technology can simultaneously track several objects with high precision but with the drawback of the line of sight that is difficult to guarantee in an interventional room. Alternatively, (ii) EM technology, whose drawback is the high sensitivity to metals plus the necessity of several wires, one for each object that needs to be tracked. Moreover, typically, EM tracking systems require choosing markers on the patient with a wand (or needle guide wire or catheter) and simultaneously selecting those markers on the preprocedural image (CT/MRI) or leveraging fiducial patches on the procedural (CT/MRI) image  [9]. Methods that do not require physical external markers have been considered [10]. However, they rely on the possibility of having 3D US images to perform the rigid registration through similarity metrics evaluation computed in volumetric patches. This approach needs further improvement for possible clinical application since physicians typically use 2D US images in the clinical environment. Another method based on similarity evaluation that considers 2D US and 3D MRI images has been developed [11]. However, it requires an initial coarse registration performed by an expert, which limits the usability scenarios of the segmentation in clinical practice. Furthermore, in the US-MRI or US-CT image fusion field of research, the effort has been made to produce a dataset for the validation of the registration method, especially from those methods mentioned above, which are based on the analysis of the US and MRI image features [12, 13].

### Skin Segmentation

Skin reference has demonstrated great relevance in many other applications such as on breast tissue, vessels and blood evaluation [14–17] or in the neurological field for surgical navigation system optimisation and registration [18, 19], as well as on the abdominal district [20, 21]. Moreover, the diffusion of 3D surface acquisition systems opens an important research branch in this direction. However, only some work includes methods for skin extraction from volumetric images, which are usually application-specific. For example, a few works segmented the skin as part of their pipeline in breast image analysis. For the diagnosis of breast diseases with the dynamic contrast-enhanced MRI (DCE-MRI), the segmentation of the breast’s first layer of skin has been obtained through a pre-processing of the image with median filters and mathematical morphology followed by the identification of the upper boundary of the breast, which is the skin boundary [16]. However, the method used to identify the upper boundary is not explicitly described and focuses only on DCE-MRI images. Another technique for breast skin identification on classical CT and MRI can be obtained through thresholding followed by morphological filters [14] or 3D vector-based connected component algorithm [15].

Thresholds have also been leveraged in other districts on the raw image [21] or after a pre-processing aimed at edge enhancement [18]. These works apply the thresholding method on the whole image to classify the pixel in the background (black) or body (white) and then use other filtering methods to clean the obtained result. Skin segmentation is generally applied to CT images, as the corresponding skin’s Hounsfield Unit (HU) value is known [21] and cannot be applied directly to other imaging modalities. In [19], the Watershed transform from markers has been used to a gradient image containing light to do dark transitions obtained from T1-MRI. Graph-based techniques have been applied on 3D US images [22] while other works can be found in 3D human reconstruction [23]. In deep learning, previous work [20, 24] focused on body composition analysis, segmenting the image into different body structures, including subcutaneous adipose tissue and the external skin edge. In [25], a combination of the Canny filter, the selection of boundaries, and a local regression has been applied to delimitate the different skin layers in 3T MRI with T2-weighted sequence. All these works have developed skin segmentation as part of their work pipelines, thus focusing on one imaging modality and leveraging the properties of that specific image.

### 3D Rigid Registration

3D rigid registration refers to aligning two 3D surfaces or point clouds. The *Iterative Closest Point *(ICP) algorithm [26] iteratively searches the closest points between two point clouds and computes a rigid transformation to align them. The *Robust Point Matching *(RPM) [27] applies a probabilistic approach to estimate the correspondences between points in two 3D point clouds. It is less sensitive to noise and outliers than ICP. The *Coherent Point Drift *(CPD) [28] applies a Gaussian mixture model to model the probability distribution of the point clouds. It supports rigid and non-rigid deformations and is more versatile than ICP and RPM. Deep learning methods, such as PointNet [29, 30] and PointNet+ [30], apply neural networks to learn features from 3D point clouds and perform registration. Deep learning methods are highly efficient regarding the time required for registration after the training, thus valuable for real-time applications. However, learning methods must be trained on large data to avoid biases, and having large and various data sets in medical applications is still a challenge.

## MR & US Fusion System

The novel co-registration is divided into two main software components integrated into the image fusion system through two executable files. The first aims to segment the external skin surface of patients acquired by MR/CT, while the second seeks to co-register the segmented skin surface with the 3D surface obtained by the camera. In this way, the integration is possible by leveraging the computer of the US system.

### Skin Segmentation

The segmentation of the 3D surface representing the patient’s skin is used to bridge the seamless integration of the heterogeneous data sources involved in the system. Indeed, extracting the body surface from volumetric imaging facilitates subsequent analyses and enables the processing of lighter data. The segmentation of the external body surface is computed according to the Hounsfield value (CT) or intensity level (MR), set as a default parameter, and represented as a triangle mesh. Given a CT/MR image paired with the skin iso-value, the proposed segmentation identifies the subject’s skin surface, which is used as input for the co-registration.

**Fig. 2:**
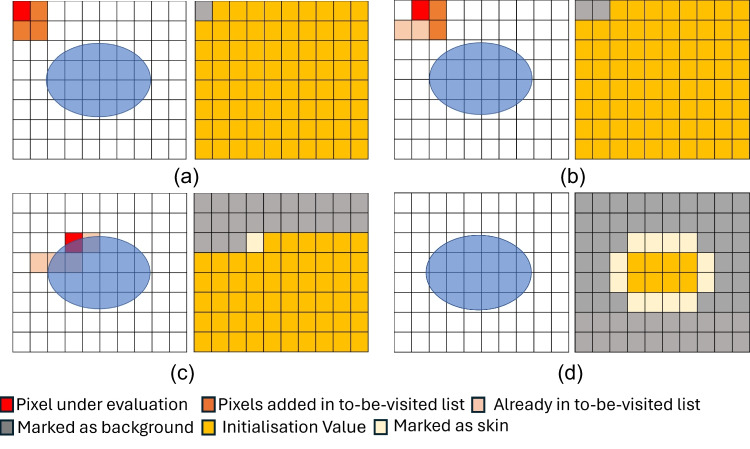
Description of the segmentation method on a slice. The toy image is on the left of each step, and the mock-up grid is on the right. **a** The algorithm’s initialisation (every pixel in the mock-up grid has a value of 2), evaluation of the first pixel, identification of the neighbourhood, and consequent assignment of background value on the mock-up grid. **b** The second step of the algorithm has the same consideration as the previous one. **c** Identification of a pixel above the threshold: the value of the mock-up pixel changes to 1 and the neighbourhood of the pixel is not inserted in the list. **d** Final segmentation

The general idea is to leverage the differences in intensities between the air and the body surface. The segmentation proceeds one slice at a time, starting from a background pixel. Then, the growth of the background region proceeds iteratively based on the pixels’ adjacency and stops when it encounters a pixel whose grey value is higher or equal to the iso-value of the skin. Through this region-growing algorithm, the evaluation expands only where the air is present; the body will be segmented as a whole object by exclusion. Algorithm 1 describes a slice-by-slice method for segmenting anatomical structures within medical images. A mock-up grid for the CT/MR image is generated, aligning with the original image’s dimensions. Initialisation involves assigning a predefined initial value to all grid elements (e.g., 2). Subsequently, the algorithm proceeds slice by slice. A starting pixel, typically situated at the corners of the slice to represent the background, is selected, and its intensity is compared against a predetermined skin isovalue (Fig. 2a). Pixels are evaluated based on their intensity level. Those below the skin isovalue are marked as background by assigning to the mock-up grid correspondent element a value of 0 and considering their neighbour as pixels to be visited in the following iteration. Pixels above the skin isovalue indicate the body edge and, thus, are marked with a mock-up grid value of 1 (Fig. 2b), and their neighbour is not considered in the following iteration. The iterative process continues by updating the mock-up grid while avoiding duplicate evaluations. Figure 2c). Upon completion, the mock-up grid delineates background (0), body edge (1), and initial value (2) regions (Fig. 2d).

**Algorithm 1:**
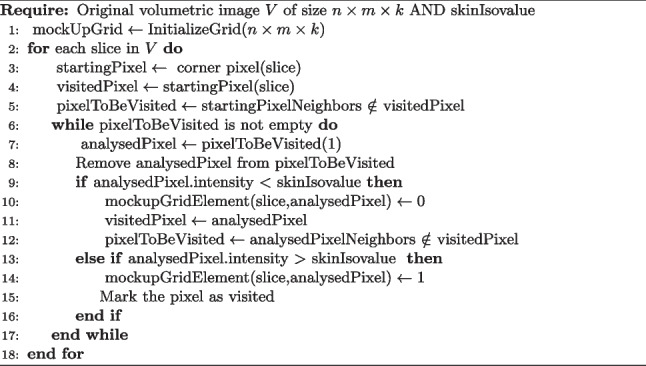
Slice-by-slice skin segmentation algorithm

Then, the segmented volume undergoes the marching cube algorithm [31] to extract a 3D surface mesh of the segmented skin, representing the input for the co-registration phase to match the MRI and the 3D surface acquired by the camera. To improve the segmentation, we add padding around each slice coloured with the minimum value appearing in the image to guarantee that it will be considered background. In this way, the algorithm proceeds through the padding pixels toward the slice’s end. The padding is helpful in case the MR/CT bed has been acquired with the patient. To reduce the overall computational time for skin segmentation, we can sub-sample each slice and each set in the case of high-resolution MR/CT images. The intensity value for the skin, i.e. the iso-value needed as an input parameter to the segmentation algorithm, is easily retrievable according to the specification of the MR/CT acquisition machine. Indeed, each manufacturer usually has standard values for each imaging modality.

### Skin Co-registration

To align the MR/CT image with the US probe, the 3D surface acquired by the camera, which is in the same reference system as the US probe and of the magnetic tracking, is rigidly co-registered with the patient skin segmented from the MR/CT images. We use rigid registration to prevent the introduction of deformations, as the two surfaces utilised for coregistration exhibit inherent differences. Specifically, these surfaces vary in connectivity and vertex count due to their distinct generation methods: the image-derived surface results from segmentation, whereas the 3D camera surface is extracted using a depth camera. The output of the co-registration is a translation vector and a rotation matrix, which co-register the segmented surface to the 3D surface acquired by the camera (i.e., the Intel RealSense in the experimental setup), minimising the corresponding misalignment. The co-registration takes the segmented surface extracted from the anatomical images (MRI/CT), the 3D surface acquired by the camera, and a reference virtual landmark as inputs. The 3D surface must be acquired by the 3D camera with a frontal view, following the guidance provided by the camera to minimise acquisition errors. The segmented surface is oriented consistently (i.e., head-feet, right-left) to avoid errors related to body symmetries.

**Fig. 3:**
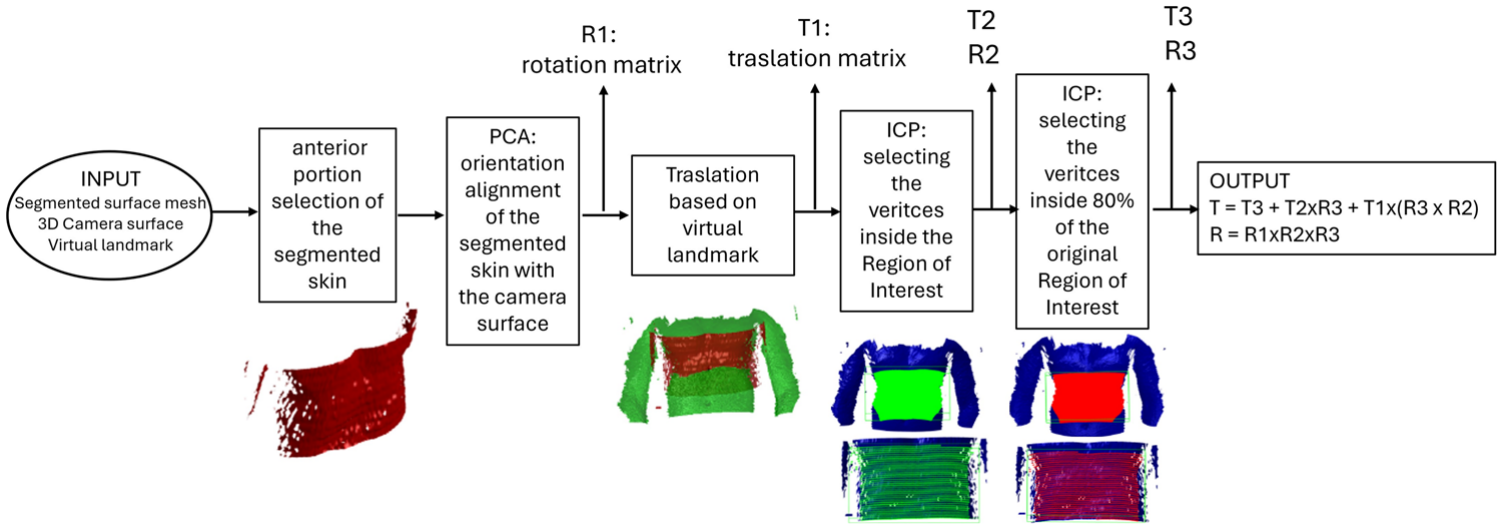
Co-registration pipeline. The selected anterior portion of the segmented surface. PCA alignment and translation on the reference points. Surface sub-regions for the first ICP run. Surface sub-regions tuning for the second ICP step (only $80\%$ of the surface considered in the first ICP run is considered in the second refinement run)

The operator manually selects one corresponding landmark point on each input surface to align the two surfaces through the US system interface (i.e., the US system monitor). Thus, the landmark is exclusively virtual and does not require any external physical placement; however, it is necessary to maintain the generality of the registration method. Indeed, certain regions of the human body, like the abdomen, do not present relevant morphological features that could be leveraged for automatic preliminary alignment. A pipeline composed by orientation adjustment through *Principal Component Analysis*(PCA), surface sub-region selection and tuning (region of interest), and various coregistration refinements leveraging the *Iterative Closest Point algorithm *(ICP [32]) allows for accurate alignment of the segmented surface with the surface acquired by the camera (Fig. 3). Then, the computed roto-translation is applied to the volumetric data to put the MR/CT images in the same reference system of the US probe, thus enabling the fusion of the MR/CT image with the US image since the tracking system tracks both the 3D camera and the US probe.

This result will allow the radiologist/surgeon to navigate the MR and US images simultaneously during the US examination or preoperatively. To optimise the time to rigidly register the segmented surface with the 3D surface acquired by the camera, the segmented surface is cut to get only the front part of the body. This way, the relevant part of the segmented surface undergoes the registration. To cut the surface, we consider the angle between the normal at each surface vertex and the sagittal axis: the vertices associated with an angle smaller than 90°are selected as part of the front surface.

**Fig. 4:**
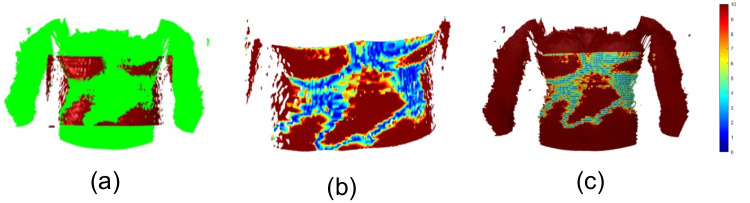
**a** Co-registration pipeline result. Error distribution **b** on the segmented surface and **c** on the camera mesh. The unit measure of the colourmap is mm

Visualising the registration error between the segmented and acquired skin (Fig. 4) gives valuable insight to assess whether acquiring a more accurate surface from the camera is necessary to improve the co-registration or if the (co-registration) results are accurate enough. The co-registration error between the segmented skin and the skin acquired by the camera is computed as the Hausdorff distance between the co-registered surfaces. Calling the segmented surface $\textbf{X}_{1}$ and the 3D surface acquired by the camera  $\textbf{X}_{2}$ we identify co-registration error by computing their Hausdorff distance $d(\textbf{X}_{1},\textbf{X}_{2}):=\max \{d_{\textbf{X}_{1}}(X_{2}),d_{\textbf{X}_{2}}(X_{1})\}$, where $d_{\textbf{X}_{1}}\left( \textbf{X}_{2}\right) :=\max _{\textbf{x}\in \textbf{X}_{1}}\left\{ \min _{\textbf{y}\in \textbf{X}_{2}}\left\{ \left\| \textbf{x}-\textbf{y}\right\| _{2}\right\} \right\}$. The minimum distance is calculated using a Kd-tree structure. Higher co-registration errors present a higher Hausdorff distance. The distance distribution is mapped to RGB colours, and each vertex is assigned the corresponding colour according to its distance from the other surface. To better analyse the distance distribution in the relevant portion of the surface, vertices that present a distance equal to or higher than 5 mm are coloured red. Vertices that have a null distance are shown in blue. The other distances are mapped to the shades between red and blue. If the error is located in areas relevant to the structure under analysis, it may prompt reconsideration of the data acquisition process. Conversely, if errors are primarily present in regions not critical to the examination, the medical professional can confidently proceed with analysing the fused MR/CT and US images.

## Experimental Results and Validation

We discuss the results on skin segmentation (see the “Skin Segmentation Results’’ section), the robustness of the skin co-registration to noise and selected parameters (e.g., HU value, virtual landmark), and the accuracy of the image fusion (see the “Co-registration Validation’’ section). We also describe the co-registration proof of concept on real subjects.

### Skin Segmentation Results

**Fig. 5:**
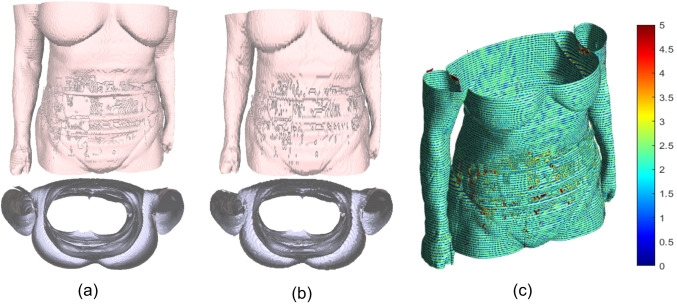
Robustness to image subsampling of the skin segmentation method. The skin surface was extracted from the segmentation of the input image at **a** high resolution and **b** low resolution. **c** Distance distribution between the two surfaces; the colourmap scale goes from 0 mm (blue) to 5 mm (red)

The segmentation (i.e., the voxel labelling) and mesh extraction have a computational cost linear to the number of voxels composing the volumetric image. Table 1 reports the timing of each algorithm step on an 11th generation Intel(R) Core(TM) i7-11700k 8 core. To better integrate the approach with existing clinical workflow, we tested its robustness to subsampling. Indeed, given the computational cost property of the method, even a light subsampling could drastically improve the performance in terms of time required for the segmentation to complete. Figure 5 shows how skin segmentation remains clean and accurate given an image and its subsampled version. We subsampled a volume image by a factor of two in each direction. The only difference is in the resolution of the surface, which is a direct consequence of the lower resolution of the subsampled original image. To confirm the maintained accuracy of the segmentation at different image resolutions, we computed the distance distribution between the surfaces extracted from a volume image and its subsampled version. In this case, the higher distances correspond to those slices and pixels missing in the subsampled version of the image, and the value of the distance is coherent with the changed dimension of the voxels. Contrary to AI methods, 3D skin segmentation does not require any training. Consequently, it does not require a large data set or various acquisitions of diverse imaging modalities, contributing to the method’s generality. The skin segmentation has been designed to be as general as possible regarding the anatomical area scanned (e.g., head, breast, total body, and abdomen) and the acquisition modality (e.g., MR, CT, PET).

**Table 1:** Computing time of 3D skin segmentation algorithm on various anatomical districts and imaging modalities

**Imaging Modality**	**Volume size**	**District**	**Volume reading**	**Segmentation**	**Skin extraction**
MRI T1	$260\times 52\times 72$	Abdomen	3 s	28 s	4 s
MRI T2	$184\times 256\times 30$	Abdomen	1 s	5 s	0.2 s
MRI	$384\times 384\times 50$	Breast	3 s	45 s	3 s
MRI T2	$384\times 384\times 46$	Breast	2 s	42 s	2 s
CT	$512\times 512\times 247$	Whole body	2 s	304 s	16 s
CT	$256\times 256\times 160$	Head	1 s	46 s	1 s

**Fig. 6:**
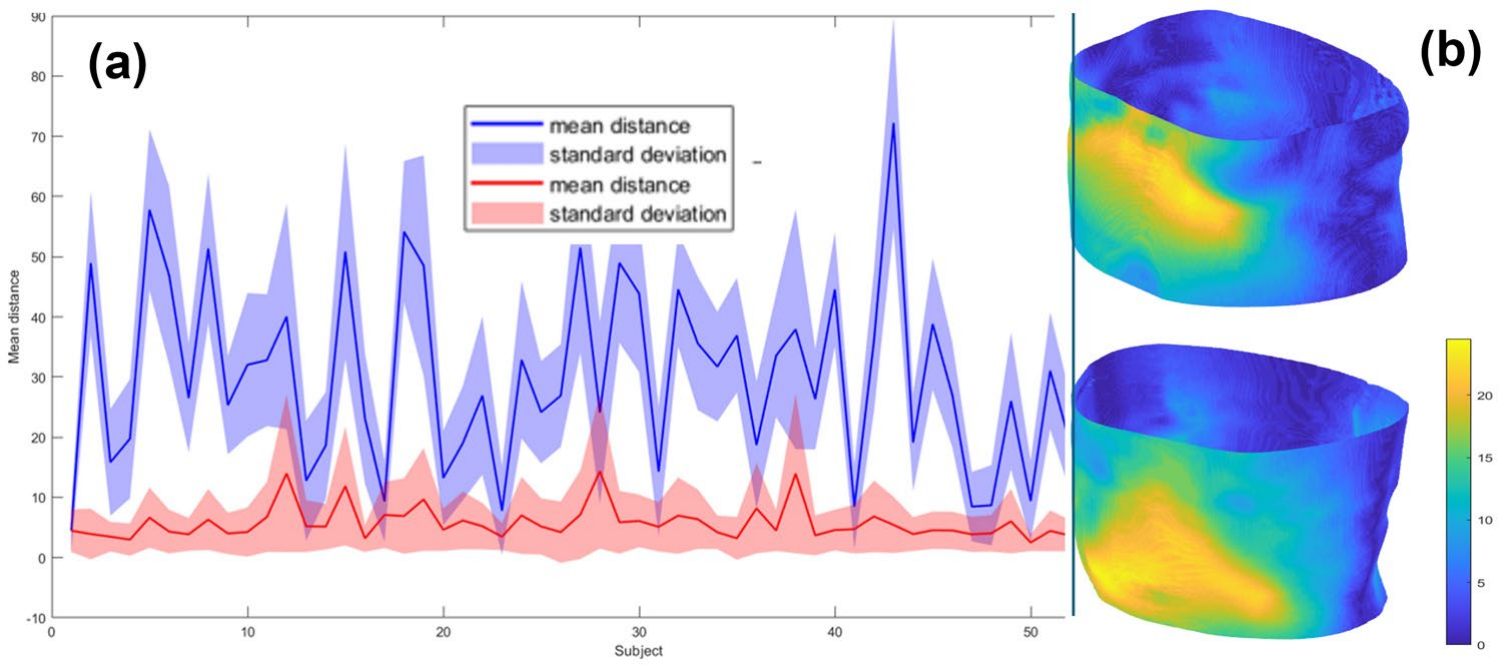
**a** Mean distance between the skin surface extracted from the CT and the surface extracted from the MRI of the same subject (red) and of different subjects (blue). **b** Distance distribution between the skin in the inhale and exhale phase extracted from CT images. (Left) Breathing movement is concentrated in the higher abdomen/lower chest. (Right) breathing movements concentrated in the abdomen. The unit measure of the colour map is mm

We tested the segmentation algorithm on the dataset by Zöllner [33], which contains 52 subjects, and for each subject, the dataset provides CT in the inhale phase, CT in the exhale phase, MR in the inhale phase and MR for the exhale phase. We tested the consistency of the skin segmentation through the differences between the obtained skin surfaces of the same subject in the different acquisition modalities. The results obtained (Fig. 6a) highlight the accuracy of the segmentation, which is entirely patient-specific. Indeed, the mean of the distance distribution between two surfaces of the same subject from different scans is highly inferior compared to the differences between surfaces obtained from various subjects. We evaluated the changes in the extracted surfaces from the same dataset between the inhale and exhale phases (Fig. 6b). The measured changes underscore the skin segmentation’s ability to align with differences in morphology of the same subject, showing which part of the abdomen and chest are more involved in breathing movements.

### Co-registration Validation

The US/MR image fusion accuracy was tested on phantoms and volunteers for system validation. The tests performed on phantoms and volunteers have been executed by technicians of the US factory, with varying experience levels.

#### Acquisition Protocol

During the ultrasound examination, the volumetric image of the patient, whether it is from a CT or MRI scan, will be loaded into the system. When capturing the external surface of the patient with the 3D camera, the patient will assume the same position held during the acquisition of the volumetric image. If possible, the patient will also be requested to inhale or exhale according to the respiratory phase of the volumetric image. Due to the speed of surface acquisition with the 3D camera, this should be fine for the patient. Once the camera acquires the surface, the patient can resume normal breathing. The virtual landmark is selected through the US system interface (i.e., the touch screen already on the US machine), and the coregistration automatically starts. At the end of the process, the sonographer can proceed with the US examination, visualising the US and volumetric images simultaneously or overlaid.

#### Phantom Tests

**Fig. 7:**
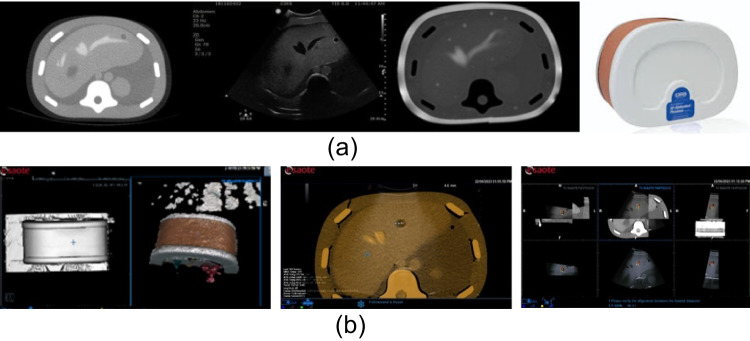
**a** MRI image of a phantom of the abdominal district. **b** From left to right: Phantom surface acquired at 0 degrees, image fusion results with CT, and accuracy error 4.3 mm

**Fig. 8:**
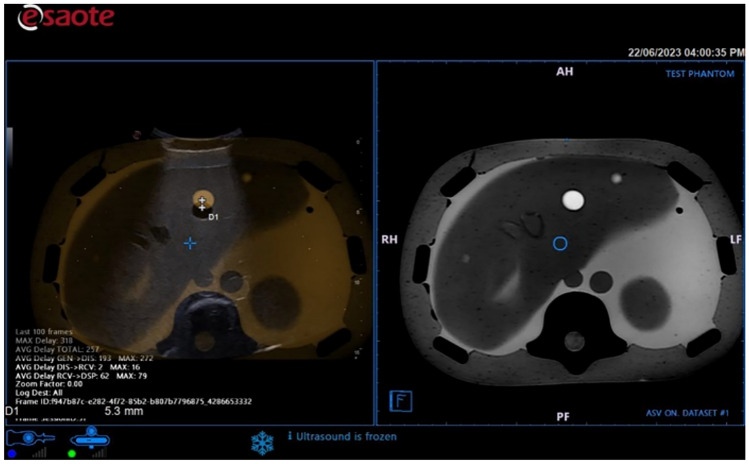
US co-registration with an MR: accuracy error 5.3 mm. The skin surface has been acquired at 0 degrees

The phantom tests have been conducted on the CT (Fig. 7) and MR (Fig. 8) images. The skin surface has been acquired by segmenting the CT acquisition of the phantom. Firstly, the 3D surface obtained by the camera was captured by moving the camera around the phantom CIRS Model 057 to simulate a better possible clinical configuration, where the EM transmitter and camera are placed around the patient bed. The accuracy result is better in the 0 degrees and 180 degrees with respect to the lateral one (90–180). In the worst-case scenario, the accuracy error varies from 4.3 to 13 mm. To further test the co-registration, we compared the results on two phantoms representing the abdominal district: CIRS Model 057 and the Kyoto Kagaku dual modality human abdomen. The first phantom is remarkably symmetric (cylindric) and does not present the anatomical morphology of the abdominal and chest region. The second is more representative of the external morphology of the area. The skin surface was acquired by segmenting the CT acquisition of the phantoms. The co-registration is accurate, and the error localises in those regions where the morphology of the surfaces differs due to the different resolutions of the MR and 3D cameras. Other sources of error are related to the acquired area’s limited dimension and symmetric shapes. The physician can correct minor errors through manual tuning to produce a more accurate result (if needed).

**Fig. 9:**
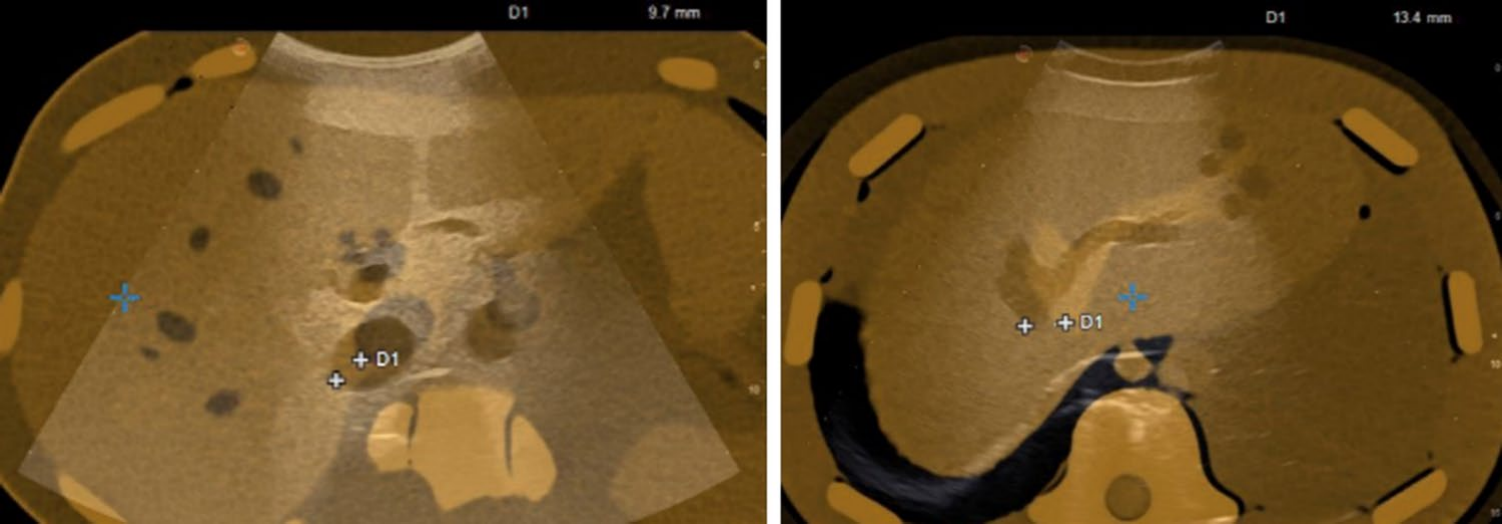
Landmark point selection on the target image (US) and registered image (MRI). The White cross corresponds to the selected landmark

The error was evaluated inside the volume using the target registration error (TRE): $\text {TRE} = \frac{1}{N} \sum _{i=1}^{N} \Vert \text {landmark}_i^{\text {registered}} - \text {landmark}_i^{\text {target}} \Vert$, where *N* is the number of corresponding landmarks, $landmark_{i}^{registered}$ is the $i^{th}$ landmark point in the registered image (i.e., the MRI),  $landmark_{i}^{target}$ the $i^{th}$ landmark point in the target image (US), and $\Vert .\Vert$ denotes the Euclidean distance. We consider one correspondent target point ($N=1$), placed by the technician on clearly visible anatomical features while visualising the US image and the MRI/CT superimposed. The system automatically computes the Euclidean distance through a standard measuring feature in the US software (Fig. 9)

##### Distance from the 3D Camera

**Fig. 10:**
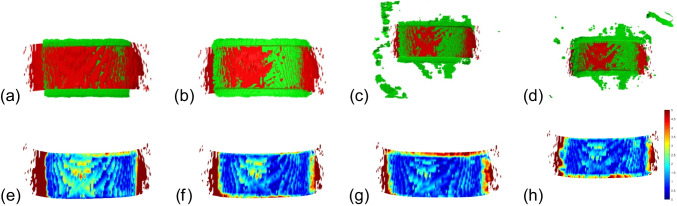
Co-registration and error distribution on a phantom acquired by a 3D camera at different distances: **a**, **e** 20 cm, **b**, **f** 25 cm, **c**, **g** 30 cm, **d**, **h** 35 cm. The error visualisation scale in the colour map goes from 0 mm (blue) to 5 mm (red)

To verify the robustness of the co-registration, the skin surface was captured by placing the 3D camera at different distances from the skin (Fig. 10). The co-registration remains stable against the acquisition distance. At high distances, the noise acquired by the camera increases, confirming the algorithm’s robustness to the noise and symmetries (Table 2).

**Table 2:** Coregistration error evaluation on phantoms

**Test**	**TRE**	**TRE**
	**Kyoto Kagaku**	**CIRS Model 057**
**Distance from camera**		
30 Cm	6.2 mm	9.6 mm
35 Cm	8 mm	8.7 mm
40 Cm	4.7 mm	11.5 mm
**Camera tilting**		
Perpendicular	5 mm	8.7 mm
30 Degrees	9.6 mm	16.5 mm
45 Degrees	17.5 mm	12 mm
**Marker misplacement**		
1.5 cm Horizontal	9.7 mm	13.5 mm
1.5 cm Vertical	12.7 mm	14.5 mm
1.5 cm Diagonal	9.7 mm	13.4 mm

##### Camera Tilting

We tested how much tilting the 3D camera at various angles during the acquisition would affect the co-registration. We kept the camera’s distance from the surface equal to 35 cm. Then, we tilted the camera with varying angles, starting from the camera perpendicular to the phantom surface, then tilting it 30 and then 45 degrees from the original position. Table 2 shows the TRE measured inside the volume for both phantoms.

##### Virtual Landmark Displacement

**Fig. 11:**
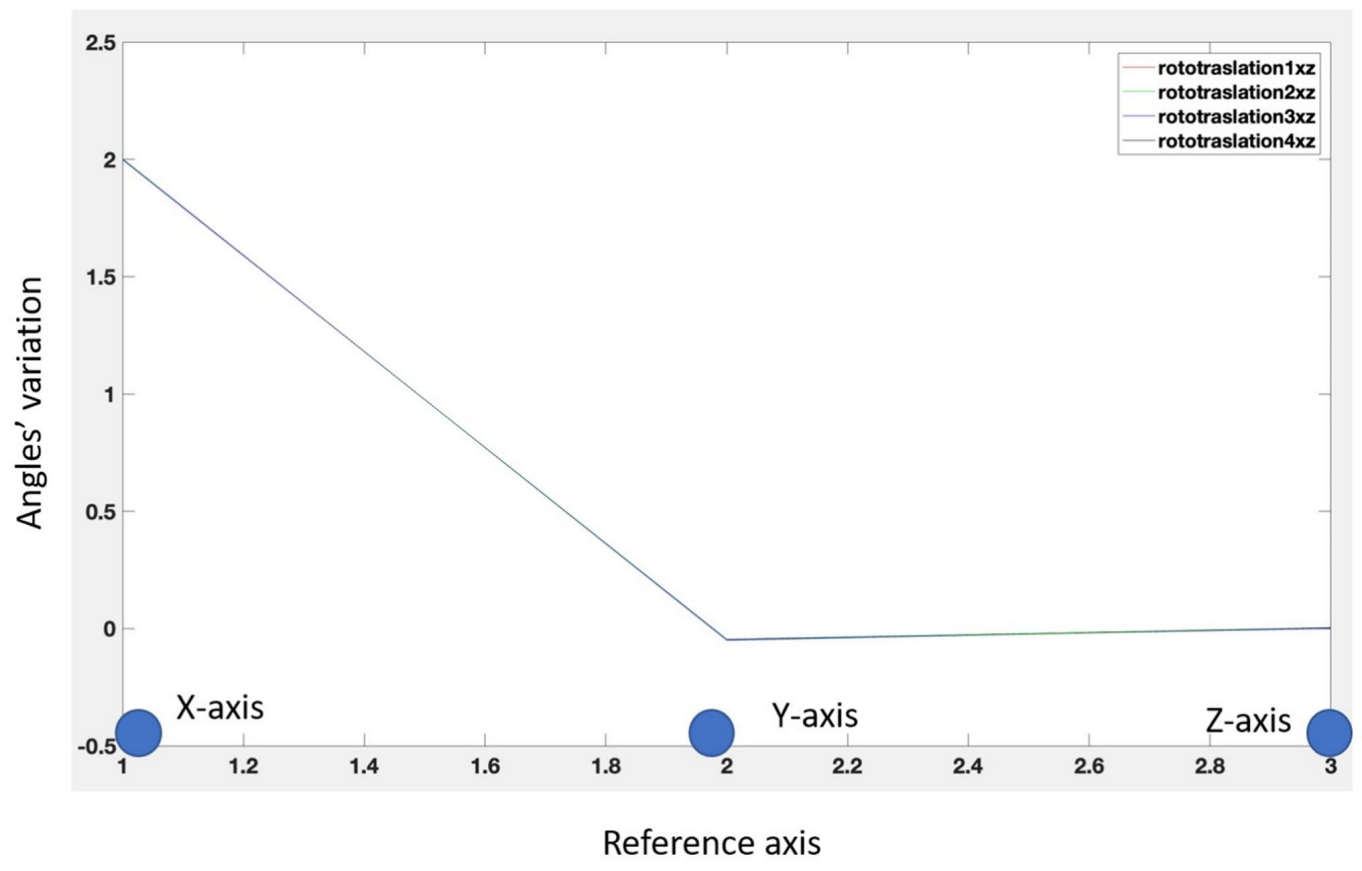
Rotation angles concerning the *X*, *Y*, and *Z* axes when a misalignment on the marker selection is present. The misalignment is in the diagonal direction (*X* and *Y*). The rotation angles remain the same (i.e. the lines overlap). Thus, the algorithm is robust to errors in the virtual landmark selection. Seme results are obtained for horizontal and vertical misalignment

To verify the influence of the selection of corresponding virtual landmarks on the co-registration, we evaluated how much the horizontal, vertical, and diagonal perturbation of the position of the virtual reference influences the fusion quality. In this experiment, the camera was fixed at 35 cm from the phantom and perpendicular to it. We select slightly displaced landmarks along the *X*, the *Z* axes, and diagonal (*X* and *Y* displacement) and measure the TRE of the fusion results (Table 2). Moreover, we measured the changes of the angles between the corresponding co-registration matrices at increasing displacements on the landmark selection; significant differences in the rotation angles were not found (Fig. 11). The robustness of the co-registration to a misplacement of the reference virtual landmarks confirms that the algorithm will not be user-dependent, i.e., the users can apply different approaches to select the landmark.

#### Volunteers Tests

**Fig. 12:**
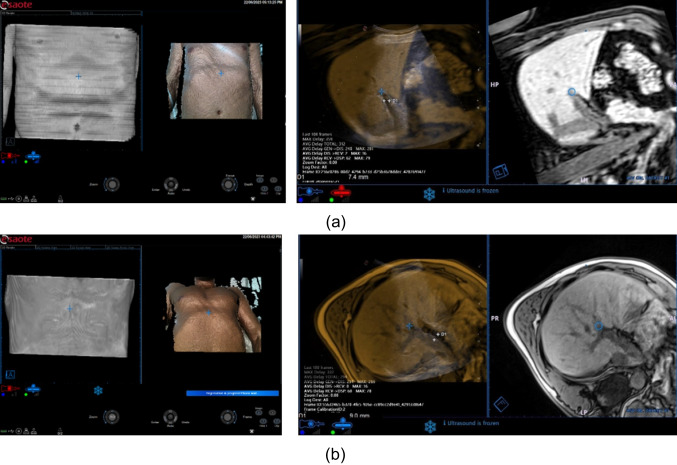
**a** Patient 1 MR images and skin surface acquired at 0-degree (left), (right) co-registration with an accuracy error of 7.4 mm, **b** Patient 2 MR image and skin surface 0-degree acquisition (left) co-registration result with accuracy error 9 mm (right)

The error in the image fusion results was evaluated in terms of TRE. The TRE error was evaluated using three volunteers, and as the anatomical reference point for error evaluation, we considered the portal vein bifurcation. Volunteer 1 has been acquired with Philips MR Serie T2 AX MVXD, and the accuracy error was 7.4mm (i.e., the distance between the portal vein bifurcation in the US and the portal vein bifurcation in the MR). Volunteer 2 has been acquired with Siemens MR Serie T2 HASTE, and the accuracy error was 9 mm. Volunteer 3 was acquired with Siemens MR Serie T2 TRUFI, and the accuracy error was 7.4mm (Fig. 12).

Our results are comparable to the ones obtained by another marker-less image fusion system based on surface coregistration by Gsaxner et al. [34]. Indeed, their mean TRE values range from 9.4 to 10.2 mm. The main difference between the two approaches is the morphology of the considered anatomical structure. They developed their method for head and neck surgery. Thus, they coregister two surfaces with morphologically relevant features. Our method obtains slightly higher error results since we get a TRE value up to 17.5 mm in the worse-case marker misplacement. However, our method applies also to surfaces that are featureless in morphological terms and are highly symmetrical.

## Conclusions and Future Work

This paper presents a method for fusing volumetric anatomical images (MRI/CT) with US images through a 3D depth sensor. The main novelty in the fusion between the two images is the co-registration between the skin surface extracted from the volumetric image and the skin surface acquired by the 3D camera. This co-registration, together with the magnetic tracking system and the 3D sensors placed on the probe and camera, allows the fusion of the MRI/CT image with real-time US acquisitions without using external physical markers. The co-registration has satisfactory accuracy and robustness to noise, virtual landmark misalignment, camera acquisition distance, and camera tilting during the acquisition phase. The error on a phantom is related mainly to the camera position, the acquired area’s limited dimension, and its symmetric shape. The error on volunteers is imputable to patient position differences between MR and US scans other than the breathing movements. The test on volunteers demonstrated that the 3D camera acquisition and co-registration streamline the fusion procedure between the US and anatomical images. This enhancement reduces the steps required for fusion imaging, making it accessible even for less experienced operators. The accuracy achieved on tests of the image fusion integrated within a US system is of the order of the millimetre. Moreover, in some tests, the MR acquisition date and the US examination were taken more than ten years apart, increasing morphological changes.

Despite the positive results obtained, our system has limitations. Indeed, it still requires the user to select a virtual landmark, which makes the overall pipeline only partially automatic. The segmentation algorithm requires the skin isovalue as an input, which is easy to retrieve but does not allow the segmentation to be fully automatic. Fast tuning is necessary to obtain a higher precision result in worst-case scenarios with errors up to 17 mm. Thus, improving the method’s accuracy could avoid this further tuning step. Even if physicians have preliminary tested the integrated system and confirmed that the time required for the overall pipeline is acceptable, future work will focus on reducing the computational time by sub-sampling the volumetric image and through the acquisition of the patient’s skin by a 3D camera with a lower resolution. Moreover, future works will focus on improving and developing the skin segmentation that currently provides promising results in accuracy and generality and its visualisation for clinical applications such as surgical intervention planning. Moreover, we will focus on an augmented system to visualise the error and represent possible misalignment in the volume image rather than on the surface.

A potential avenue for further improvement in the coregistration involves exploring camera registration while partially dressing the patient. Further enhancement could include making the image fusion system independent of the patient’s position. Finally, future refinements could consist of the management of the breathing phase with compensation methods [35] or by tracking the patient’s breathing to synchronise the two image modalities in their best match during the breathing phase  [36–39].

## Data Availability

Not applicable.
